# Dementia and suicide: What relationship to establish and what risks to consider?

**DOI:** 10.1192/j.eurpsy.2021.1130

**Published:** 2021-08-13

**Authors:** F. Andrade, R. Guedes, C. Santos

**Affiliations:** Psychiatric Service, Centro Hospitalar Universitário de São João, Porto, Portugal

**Keywords:** dementia, Suicide, Risk factors

## Abstract

**Introduction:**

Given the marked population aging in the world, the incidence of dementia has significantly increased, becoming a growing health care problem. Suicide is a considerable health issue throughout the life span, being prevalent in older adults, and in many countries the highest suicide rates are found in the elderly. Thus far, the relationship between dementia and suicide remains poorly understood and inconsistent.

**Objectives:**

The aim of this study is to do a non-systematic review of the current literature regarding the association between suicide risk and dementia.

**Methods:**

We conducted a research using the Medline database, through the Pubmed search engine, using the following key-words: “dementia”, “suicide” and “risk factors”.

**Results:**

Overall, the risk of suicide in people with dementia appears to be the same as that of age-matched general population. However, studies point to the existence of a number of factors that can increase this risk, such as: early age of dementia diagnosis, recent diagnosis, disease awareness and depression, hopelessness, male gender, failure to respond to anti-dementia medication, history of inpatient psychiatric hospitalizations, concurrent medical comorbidities.

**Conclusions:**

Studies have reported mixed results as to whether dementia itself is an independent risk factor for suicide. Despite these findings, understanding the risk factors for suicide among people with dementia is crucial and suicide prevention efforts should be carried out in this population.

